# Analgesic effects of the cathepsin K inhibitor L-006235 in the monosodium iodoacetate model of osteoarthritis pain

**DOI:** 10.1097/PR9.0000000000000685

**Published:** 2018-10-05

**Authors:** Lilian N. Nwosu, Peter R.W. Gowler, James J. Burston, Biljana Rizoska, Karin Tunblad, Erik Lindström, Urszula Grabowska, Li Li, Dan F. McWilliams, David A. Walsh, Victoria Chapman

**Affiliations:** a Arthritis Research UK Pain Centre, School of Life Sciences, Queen's Medical Centre, University of Nottingham, Nottingham, United Kingdom; b Medivir AB, Huddinge, Sweden; c Arthritis Research UK Pain Centre, Academic Rheumatology, City Hospital, University of Nottingham, Nottingham, United Kingdom

**Keywords:** Osteoclast, Pain, Joint, Rat, Osteoarthritis

## Abstract

**Introduction:**

The mounting evidence that osteoclasts play an important role in osteoarthritis (OA) pain lead us to investigate the effects of L-006235, a potent and selective inhibitor of cathepsin K, on pain behaviour and joint pathology in a model of OA pain.

**Methods:**

Effects of preventative (30 and 100 mg/kg) and therapeutic (100 mg/kg) oral dosing with L-006235 on weight-bearing asymmetry, hind paw withdrawal thresholds, cartilage and bone pathology, synovial inflammation, and drug exposure were studied in the monosodium iodoacetate rat model of OA pain.

**Results:**

Preventative L-006235 inhibited weight-bearing asymmetry from day 14, with this measure nearly abolished by the higher dose. In the same treatment setting, L-006235 prevented lowering of hind paw withdrawal thresholds from day 7. Exposure to L-006235 in plasma was higher for the 100 mg/kg dose, compared with 30 mg/kg. Therapeutic dosing with L-006235 from day 14 significantly inhibited weight-bearing asymmetry, compared with monosodium iodoacetate vehicle rats. Regression analysis revealed a significant interaction coefficient of the effects of L-006235 on weight-bearing asymmetry and synovitis score, but not for cartilage damage nor osteophyte scores.

**Conclusion:**

Our novel finding that cathepsin K inhibition is analgesic in a clinically relevant model of OA pain provides new evidence for the therapeutic potential of this target.

## Abstract 2

Supplemental Digital Content is Available in the Text.

## 1. Introduction

Osteoarthritis (OA) is the fastest growing chronic pain disease^31,46^ and is the most common form of arthritis, causing disability and reducing quality of life.^11^ Although OA was considered to be primarily a disease of the cartilage, clinical and preclinical evidence supports bidirectional interactions between cartilage and subchondral bone turnover in OA. The contribution of subchondral bone mechanisms to the pathogenesis and progression of OA is increasingly recognised.^20^

Animal models mimic key aspects of OA joint pathology and pain behaviour (see references in Ref. 44). The monosodium iodoacetate (MIA) model is associated with pain behaviour on weight-bearing, lowering of distal pain pressure threshold, synovial inflammation, cartilage damage, and subchondral bone remodelling including increased osteoclast number and numbers of osteochondral channels and osteophyte formation.^47^ Numbers of subchondral bone osteoclasts are significantly and positively associated with pain behaviour in the MIA model.^37^ Cathepsin K is a cysteine protease predominantly expressed by osteoclasts and involved in bone resorption,^3^ under normal physiological conditions; this enzyme degrades bone matrix proteins, such as type I collagen. In OA, cathepsin K also degrades the main components of cartilage matrix, type II collagen and aggrecan.^7^ Expression of cathepsin K and the osteoclast marker TRAP is increased in human OA bone.^27^ Cathepsin K mRNA and protein expression are increased in synovial fibroblasts and CD68^+^ macrophages in human OA samples,^8,25^ and expression in chondrocytes is increased in relation to OA severity.^23^

Overexpression of cathepsin K leads to spontaneous synovitis and cartilage degeneration,^30,33^ and this enzyme has been strongly implicated in the degradation of articular cartilage in a naturally occurring equine model of OA.^43^ Inhibitors of cathepsin K attenuated lesion severity and biomarkers of collagen degradation in a canine model of OA.^6,26^ Similarly, significant protective effects of cathepsin K inhibition on subchondral bone integrity were reported in a rabbit model of OA.^14,26^ Although effects of cathepsin K inhibition on OA pain behaviour have yet to be reported, such a treatment reduced the mechanosensitivity of knee afferent nerve activity in a guinea pig model of spontaneous OA,^29^ suggesting that cathepsin K inhibition might reduce aberrant OA pain.

The aim of this study was to evaluate the effects of inhibition of cathepsin K on pain behaviour and joint pathology in a rat model of OA. L-006235 is a potent and selective cathepsin K inhibitor with oral bioavailability and more than 5000-fold selective for cathepsin K vs cathepsins B, L, and S.^32^ L-006235 is potent against rat cathepsin K with an IC_50_ of 7 nM. Previously, prophylactic L-006235 reduced histological markers of disease severity in a murine model of inflammatory arthritis.^41^ In this article, we have used the MIA model of OA pain to investigate the effects of L-006235 on the development of pain behaviour, joint pathology, and a spinal marker of central sensitization in the rat. Separately, the ability of L-006235 to reverse established pain behaviour in this model of OA joint pain was investigated.

## 2. Methods

### 2.1. Animals

All animal experimental procedures conducted in the United Kingdom were in accordance with UK Home Office Animals (Scientific Procedures) Act (1986) and were in line with the ARRIVE guidelines.^21^ Experiments were conducted in a blinded fashion. Adult male rats (96 Sprague–Dawley rats, weight 180–200 g; Charles River, Margate, United Kingdom, group housed 4 per cage) were housed in temperature-controlled (20–22°C) rooms in conventional cages under a 12-hour light–dark cycle, lights on at 7 am, and off at 7 pm. Rats were allowed free access to standard rodent chow and water throughout the day, apart from a fasting period 4 hours before blood collections.

All animal studies performed at Medivir AB were in accordance with relevant guidelines and regulations provided by the Swedish Board of Agriculture. The ethical permissions were provided by an ethical board specialized in animal experimentation (Stockholm South Animal Research Ethical Board). Adult male rats (6 Sprague–Dawley rats, 8 weeks of age; Charles River, Sulzfeld, Germany) were housed (max 4 animals/cage) in conventional cages under a 12-hour light–dark cycle. Rats were allowed standard rodent diet and water ad libitum, except for a fasting period overnight (from 17:00 in the evening to 11:00 in the morning) before dosing with compound and for an additional 3 hours after dosing (in total 18 hours of fasting).

### 2.2. Intra-articular injections and behavioural testing

Intra-articular injection of MIA is associated with joint pathology^12,18^ and pain behaviour^4,22,37^ comparable with clinical OA. Investigators were blinded to the model (except in the therapeutic study where all rats received intra-articular injection of MIA) and the treatments at all stages of the study. Rats were randomly allocated to treatment groups, and then matching of control measures was performed to ensure that groups were balanced. All rats were habituated to testing area and equipment for 1 day before any procedures were conducted.

Rats were briefly anesthetized (isoflurane 2.5%–3% in 100% O_2_), and once areflexic received a single intra-articular injection of 1 mg MIA (Sigma, Gillingham, Dorset, United Kingdom) in 50-μL saline or 50-μL saline through the infrapatellar ligament of the left knee.^37^ Pain behaviour was assessed at baseline before injection (day 0) and then after intra-articular injection twice weekly. Weight distribution on the left (ipsilateral) and right (contralateral) hind limb was assessed using an incapacitance tester (Linton Instrumentation Diss, Norfolk, United Kingdom) as previously described.^37^ Changes in hind paw withdrawal thresholds (PWTs) were assessed using von Frey monofilaments; Linton Instrumentation, Norfolk, United Kingdom (Semmes-Weinstein monofilaments of bending forces 0.4–26 g) as previously described.^37^

### 2.3. Formulation of L-006235

L-006235 was synthesized by GVK Bio (Hyderabad, India) and formulated in 20% hydroxypropyl-beta-cyclodextrin (HP-β-CD) in water at concentrations of 3 and 10 mg/mL.

### 2.4. Administration of L-006235 in the monosodium iodoacetate model

**Figure 1.**
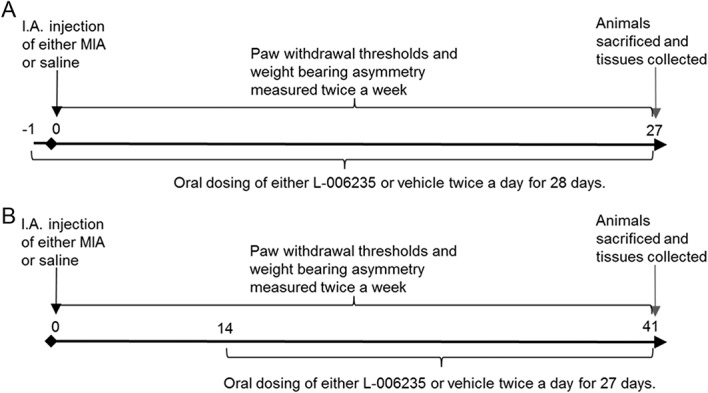
Study design timelines. (A) Timeline for the 2 independent preventative dosing studies. From day −1, rats received either vehicle or L-006235 (twice daily). Rats received intra-articular (I.A.) injection of 1-mg MIA or saline into the left knee joint on day 0, and pain behaviour was quantified at baseline and twice weekly. On day 26, rats received the final dose of treatment, and 24 hours later, final pain behaviour testing was conducted, and rats were humanly euthanized. (B) Timeline for the therapeutic dosing study. Rats were dosed with either L-006235 or vehicle for 27 days starting from 14 days after MIA injection.

In the first study, the effects of preventative treatment with 30-mg/kg L-006235 were studied. In the second study, the effects of preventative treatment with 30-mg/kg and 100-mg/kg L-006235 were studied. Rats received either vehicle (20% HP-β-CD) or L-006235 starting 1 day before MIA injection. L-006235 was administered orally twice a day for a total of 28 days (day −1 to 27 after MIA injection). Pain behaviour was measured at baseline before MIA injection and then twice a week until day 27. Pain behaviour was measured before the first dose of the day and was measured 24 hours after blood sampling (see below). In the third study, L-006235 was given once pain behaviour was established in the MIA model (therapeutic protocol). In this case, all rats received oral treatment with vehicle (twice daily) for the first 14 days after intra-articular injection of MIA. On day 14, half of the rats continued receiving vehicle and the other half received L-006235 (100 mg/kg) twice daily for 27 days (day 14–41 after MIA injection). As with the preventative study, pain behaviour was measured before the MIA injection and then twice a week until day 41 after injection. Similarly, behaviour was measured before rats received the first oral dose each day and was measured 24 hours after blood sampling (see below). See Table 1 for group sizes and Figure 1 for further study design.

**Table 1:**
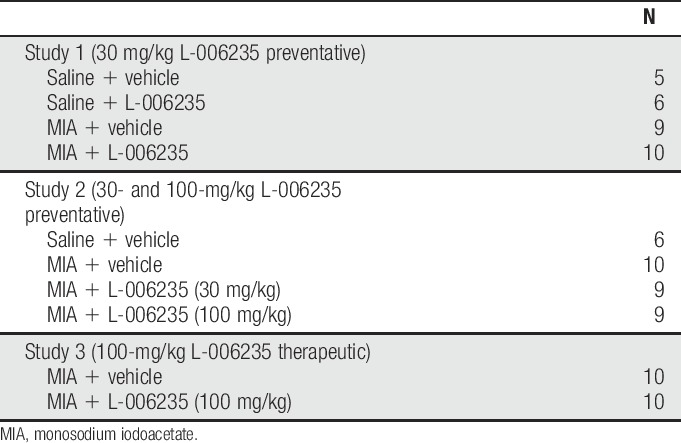
Group sizes for in vivo studies.

### 2.5. Blood collection

Blood was collected from the L-006235–treated animals for bioanalysis of L-006235 on day 8, 22, and 27 after MIA induction in the first study, on day 9 and 27 after MIA induction in the second study and on day 40 after MIA induction in the third (therapeutic) study (app. 500 µL). Blood was collected through the tail vein at 1, 2, 3, 6, and 24 hours after the dose. In total, 1 to 3 blood samples were collected from each rat for bioanalysis of L-006235. In the first study, samples were collected on day 8 and on day 22 at 2 hours after dose, and a third sample was collected on day 27 at 24 hours after dose. In the second study, one sample was collected at 1, 3, or 6 hours after dose at the first sampling occasion (day 9), and a second sample was collected 2 hours after dose on day 27. In the third study, one sample was collected at 3, 6, or 24 hours after dose on day 40/41. Blood was collected from all animals into separate Li-heparin–coated tubes for plasma separation and placed in room temperature for at least 30 minutes and protected from light before centrifugation (10 minutes, 3600 rpm, at approximately 22°C) within 1 hour of collection. The plasma samples were immediately frozen and stored at minimum −20°C until analysis.

### 2.6. Bioanalysis of L-006235

Bioanalysis of L-006235 in rat plasma was performed using protein precipitation followed by liquid chromatography-tandem mass spectrometry detection. Plasma (10 µL) was mixed with 50 µL of acetonitrile containing 100 nM of losartan and indinavir as internal standards. The sample was centrifuged at 20,000*g* for 10 minutes at 10°C, and the supernatant was diluted 1:1 with MilliQ water. Samples (5 μL) were injected onto the liquid chromatography-tandem mass spectrometry system. The lower limit of quantification (LLOQ) was 1 nM.

Pharmacokinetic (PK) data analysis was performed using the software Phoenix WinNonlin (version 6.4; Certara). Pharmacokinetic data for L-006235 were analysed using noncompartmental methodology, and the following PK parameters were reported:

(1) The area under the plasma concentration vs time curve from time 0 to time 24 hours (AUC_0–24_) was calculated by the log/linear trapezoidal method. The last sampling time point was 24 hours after dose.
(2) Maximal plasma concentration (C_max_).
(3) Time at maximal concentration (T_max_).

### 2.7. Histological staining and quantification of knee joint pathology

On day 27 (preventative studies) or day 41 (therapeutic study), rats underwent a final pain behaviour assessment and then were euthanized with sodium pentobarbital and transcardially perfused with saline, followed by 4% paraformaldehyde (in 0.1-M phosphate-buffered saline). Tibiofemoral joints with the synovia attached were dissected and postfixed in paraformaldehyde for 48 hours and were then transferred into ethylenediaminetetraacetic acid (EDTA) solution for decalcification. Knees were then sectioned for staining with haematoxylin and eosin.^10^ The pathology scoring was performed on 3 stained sections per rat^10^ as previously described.^37^ Cartilage damage and osteophytes were scored using the Janusz method as previously described.^17^ Cartilage damage was evaluated from 1 (minimal superficial damage) to 5 (severe full-thickness degeneration to tidemark). This score was multiplied by the extent of cartilage area involved (1/3, 2/3 or 3/3) to give a maximum score of 15. Osteophytes were graded from 1 to 3 for mild (<40 µm), moderate (40–160 µm), or severe (>160 µm). Synovitis was scored to assess lining thickness and cellularity from a scale of 0 (lining layer, 1–2 cells thick) to 3 (lining layer >9 cells thick and/or severe increase in cellularity).^2^

### 2.8. Synovial immunohistochemistry

Perfusion-fixated knee joint sections from the second preventative experiment were labelled for the macrophage markers CD68 and CD206 (2 sections per rat). Sections were first dewaxed in xylene before being rehydrated in decreasing concentrations of ethanol (100%, 70%) before being incubated in distilled H_2_O. Sections were then incubated in a Tris-EDTA buffer (10-mM Tris Base, 1-mM EDTA, 0.05% Tween 20, pH 9.0) at 90°C for 20 minutes. After returning to room temperature, sections were washed in 0.05M Tris-buffered saline (TBS) before being blocked for 10 minutes with a peroxidase blocking solution (BLOXALL, Vector Labs, Burlingame, CA). Sections were washed again in 0.05M TBS before being incubated for an hour in blocking serum (made up as described in the Vectastain Elite ABC-HRP Kit [Vector Labs, PK-6200]). Sections were then incubated in either 1:400 rabbit antimannose (Abcam, Cambridge, United Kingdom: ab64693) or 1:400 mouse anti-CD68 (BioRad, Watford, United Kingdom: MCA341GA) overnight. Sections were washed in 0.05M TBS before being incubated with biotinylated secondary antibody for 45 minutes (made up as described in the Vectastain Elite ABC-HRP Kit [Vector Labs, PK-6200]). After washes in 0.05M TBS, sections were incubated in ABC reagent for 30 minutes (made up as described in the Vectastain Elite ABC-HRP Kit [Vector Labs, PK-6200]). Sections were then incubated in 3,3′- diaminobenzidine (DAB) peroxidase (Vector Labs: SK-4100) for 9 minutes. Sections were then counterstained with Harris's haematoxylin before being dehydrated in increasing concentrations of ethanol (70%, 100%). Sections were then mounted in DPX. Immunostaining was visualised using a ×40 objective (Zeiss, Oberkochen, Germany: Axioskop-50) and imaged using a camera (Zeiss: AxioCam MRc). Images used for analysis were taken from the synovial lining and sublining layers on the medial side of the joint. Images were converted to 8-bit and passed through a bandpass filter. Images were thresholded, and the number of above threshold particles was automatically counted. Counts were checked manually to ensure consistency by the automated system. The area of synovia images was calculated, and data are presented as the number of positively stained cells per square millimeter.

### 2.9. Spinal cord glial cell immunofluorescence

Astrogliosis was assessed in the dorsal horn of the spinal cord using perfused tissue from study 2: preventative 100-mg/kg L-006235. The lumbar region of spinal cords from MIA rats treated with either vehicle or L-006235 was sectioned (40-µm thick sections). Immunolabelling for glial fibrillary acidic protein (GFAP) was performed as previously described,^35^ with sections incubated with 1:100 rabbit anti-GFAP (Abcam: ab48050) overnight at room temperature. Sections were then developed after being incubated with 1:300 Alexafluor 568 conjugated goat antirabbit secondary antibody. Images were taken from the superficial laminae of both the ipsilateral and contralateral dorsal horn with a Zeiss 200M microscope using a 20 × 0.8 numerical aperture objective lens. Images were autothresholded using the Renyi entropy threshold value,^19^ and the suprathreshold areas of labelling were then quantified using ImageJ software.

### 2.10. Quantitative real-time polymerase chain reaction

A separate cohort of rats were injected with either 1-mg/50 µL MIA (n = 6) or 50-µL saline (n = 6). Twenty-eight days after injection, rats were injected with sodium pentobarbital, and the ipsilateral dorsal quadrant of the lumbar region of the spinal cord was taken for analysis. Tissue was homogenised in TriReagent, and RNA was collected (DirectZol miniprep kit; Zymo Research, Irvine, CA) according to manufacturer's instructions. After extraction, 360 ng of total RNA was reverse transcribed using AffinityScript reverse transcriptase (Agilent Technologies, Santa Clara, CA) after manufacturer's instructions. The reactions were incubated at 25°C for 10 minutes, 37°C for 50 minutes, and 70°C for 15 minutes and then cooled on ice. Expression of the cathepsin K gene was quantified as previously described.^16^ Primers and probes were designed using Primer Express v3 (Thermofisher, Waltham, MA) and synthesised by Eurofins Genomics (Ebersberg, Germany). Cathepsin K forward primer 5′- CAGCAGGATGTGGGTGTTCA -3′, reverse primer 5′- CACTGCGTGTCCAGCGTTT -3′, Taqman probe 5′- CTGCTACCCGTGGTGAGCTTTGCTCTATC -3′, β-actin forward primer 5′- AGCCATGTACGTAGCCATCCA -3′, reverse primer 5′- TCTCCGGAGTCCATCACAATG -3′, and Taqman probe 5′- TGTCCCTGTATGCCTCTGGTCGTACCAC -3′.

### 2.11. Biomarker measurements

To assess the effects of L-006235 on biomarkers of bone resorption and cartilage degradation, a single dose of L-006235 (100 mg/kg) or vehicle (20% HP-β-CD) was administered through oral gavage to a separate group of naive rats (n = 3/group), and blood (app. 100 µL) was collected at baseline (before dosing) and at 1, 3, 6, and 24 hours after dosing for biomarker analyses. Levels of CTX-I and CTX-II in rat serum were measured using commercially available kits (RatLaps [CTX-I] EIA and Serum Pre-Clinical CartiLaps [CTX-II] ELISA; IDS Nordic, Herlev, Denmark), according to the recommendations by the manufacturer.

### 2.12. Data analysis

Results are displayed as mean ± SEM. All statistics were calculated using Prism 5.0 or 7.0 software (Graphpad, La Jolla, CA). Weight-bearing data were analysed with a 2-way analysis of variance (ANOVA) with a Bonferroni post hoc test. Hind PWTs were log-transformed before analysis with 2-way ANOVA with a Bonferroni post hoc test. Differences between the area under the curve for weight bearing and the log-transformed PWTs were analysed using a 1-way ANOVA. Data were considered significant if *P* values were less than 0.05. Differences in the expression of the cathepsin K mRNA between saline and MIA-injected rats, and the differences in GFAP immunoreactivity between L-006235 and vehicle-dosed MIA rats were compared with an unpaired *t* test. The statistical interaction between weight bearing and each OA feature (cartilage damage, osteophytes, or synovitis) was examined using a product term for Treatment Group*OA Pathology. Statistical significance of the interaction term provides a measure of the association between weight-bearing pain behaviour and OA pathology, and the effect of L-006235 on this association.

## 3. Results

### 3.1. Preventative L-006235 attenuates osteoarthritis pain behaviour

**Figure 2.**
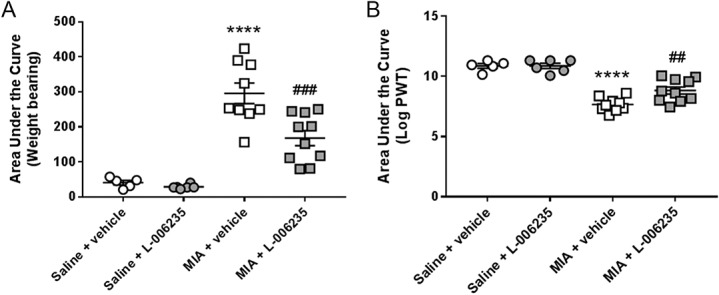
Effect of preventative L-006235 (30 mg/kg) on MIA-induced weight-bearing asymmetry (A) and log-transformed ipsilateral hind paw withdrawal thresholds (B) (study 1, Fig. 1 for study design). Group sizes were: saline + vehicle, n = 5; saline + L-006235, n = 6; MIA + vehicle, n = 9; and MIA + L-006235, n = 10. Data are area under the curve and were analysed with a 1-way ANOVA: ****P < 0.0001 saline + vehicle vs MIA + vehicle; ###*P* < 0.0001, ##*P* < 0.01 MIA + vehicle vs MIA + L-006235. Data are presented as mean ± SEM. ANOVA, analysis of variance.

**Figure 3.**
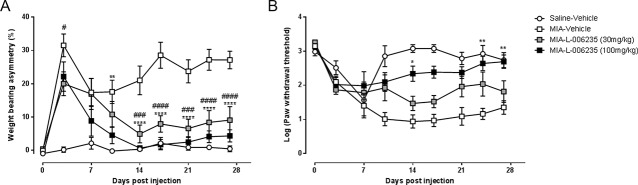
Dose-related effects of L-006235 on MIA-induced weight-bearing asymmetry (A) and log-transformed ipsilateral hind paw withdrawal thresholds (B). See Figure 1 for dosing details (study 2, Fig. 1 for study design). Group sizes were saline + vehicle, n = 6; MIA + vehicle, n = 10; MIA + L-006235 (30 mg/kg), n = 9; and MIA + L-006235 (100 mg/kg), n = 9. Data were analysed with a 2-way ANOVA with a Bonferroni post hoc test: #*P* < 0.05; ###*P* < 0.001, ####*P* < 0.0001 MIA + vehicle vs MIA + L-006235 (30 mg/kg); *P < 0.05, **P < 0.01, and ****P < 0.0001 MIA + vehicle vs MIA + L-006235 (100 mg/kg). Data are presented as mean ± SEM. ANOVA, analysis of variance.

Intra-articular injection of MIA resulted in a significant increase in weight-bearing asymmetry and a lowering of hind PWTs over the course of the model, compared with the saline-injected control rats (supplementary Figure 1, available at http://links.lww.com/PR9/A32). Two independent preventative dosing studies were performed. Twice daily oral administration of L-006235 (30 mg/kg), but not vehicle, significantly attenuated MIA-induced pain behaviour (weight-bearing asymmetry and lowered PWTs) in the first preventative study (Figs. 2A and B, supplementary Figure 1 for time course, available at http://links.lww.com/PR9/A32). In the second preventative study, effects of twice daily oral administration of 30 and 100 mg/kg L-006235 on the 2 measures of pain behaviour were compared. Time course data analysis revealed that neither dose of L-006235 altered the early peak in weight-bearing asymmetry at day 3, but both doses consistently inhibited weight-bearing asymmetry from day 14 postmodel induction onwards (Fig. 3A). The higher dose of L-006235 nearly abolished weight-bearing asymmetry, compared with the effect of vehicle treatment, at the later time points in the study (Fig. 3A). L-006235 did not alter the initial drop in hind PWTs but did prevent any further lowering in thresholds at the later time points from day 7 onwards (Fig. 3B). There was a clear dose-related effect of L-006235 for both measures of pain behaviour, which was paralleled by a higher plasma exposure after oral administration of the higher dose of L-006235 (100 mg/kg), compared with the lower dose.

**Figure 4.**
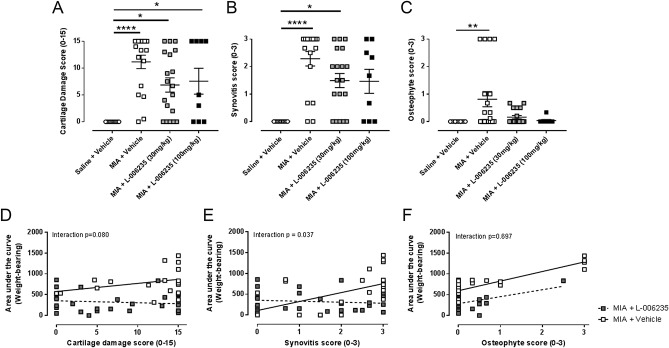
(A–C) Effects of preventative L-006235 on MIA-induced cartilage damage (A), synovitis (B), and osteophyte score (C). See Figure 1 for study design. Statistical analysis with a Kruskal–Wallis test and Dunn's post hoc test: *P < 0.05, **P < 0.01, ****P < 0.0001 vs saline + vehicle. Group sizes are as follows: saline + vehicle, n = 11; MIA + vehicle, n = 18; MIA + L-006235 (30 mg/kg), n = 19; and MIA + L-006235 (100 mg/kg), n = 9. Data are presented as mean ± SEM. (D–F) Weight-bearing plotted against cartilage damage (D), synovitis (E), and osteophyte score (F). The univariate lines of best fit for MIA + vehicle (solid line) vs MIA + L-006235 (dashed line) (both doses). The statistical interaction between weight bearing and each OA pathology feature was examined using a product term for Treatment Group*OA Pathology. Statistical significance of the interaction term implies that the measured association between weight-bearing pain behaviour and cartilage damage (D) and synovitis score (E) was changed by L-006235.

To ascertain whether the effects of L-006235 on pain behaviour were related to effects on joint damage, histological analysis was performed. Intra-articular injection of MIA was associated with significant cartilage damage, synovitis, and osteophyte score at the end of the study (Fig. 4). Neither dose of L-006235 significantly altered the 3 parameters of joint damage studied (Figs. 4A–C, supplementary Figures 2A and B, available at http://links.lww.com/PR9/A32). However, after treatment of MIA rats with the higher dose of L-006235, synovitis scores were not significantly different compared with saline vehicle-treated rats (Fig. 4B). The higher variability in the osteophyte score in MIA vehicle-treated rats reduced the opportunity to detect any potential effects of L-006235 on this parameter (Fig. 4C). To understand the potential relationship between effects of L-006235 on pain behaviour and joint pathology, a regression analysis was performed. There was a significant interaction coefficient of the effects of L-006235 on weight-bearing asymmetry and synovitis score, but not for the cartilage damage or numbers of osteophytes (Figs. 4D–F), suggesting that the positive association between synovitis score and weight-bearing asymmetry has been weakened by L-006235.

To further explore the effects of L-006235 on synovial inflammation, immunohistochemistry analysis of the numbers of macrophages in the synovium was performed. There was a nonsignificant increase in the number of CD68^+^ cells in the synovium 27 days after MIA injection when compared with saline-injected rats (supplementary Figure 2C, available at http://links.lww.com/PR9/A32). In the group that received preventative L-006235, the number of CD68^+^ cells was lower compared with vehicle-treated rats (supplementary Figure 2A, available at http://links.lww.com/PR9/A32). The mannose receptor, CD206, has been used as a marker for macrophages that have a reparative phenotype.^42^ There was a nonsignificant increase in the number of CD206^+^ cells in the synovium of rats with MIA-induced joint pain, compared with saline controls (supplementary Figure 2D, available at http://links.lww.com/PR9/A32); however, there was no significant effect of L-006235 on this cell count.

To investigate any potential effects of L-006235 on spinal pain processing, expression of cathepsin K mRNA in the spinal cord was quantified. Cathepsin K mRNA was expressed to a comparable extent in the dorsal horn of the spinal cord in saline and MIA-treated rats (supplementary Figure 3A, available at http://links.lww.com/PR9/A32). Glial fibrillary acidic protein immunofluorescence in the dorsal horn of the spinal cord is a marker of mechanisms of central sensitization and is significantly increased in the MIA model of OA pain.^35^ Preventative L-006235 (100 mg/kg) did not alter GFAP immunolabelling in the dorsal horn of the spinal cord of rats 27 days after intra-articular injection of MIA (supplementary Figure 3B, available at http://links.lww.com/PR9/A32).

### 3.2. Therapeutic L-006235 attenuates osteoarthritis pain behaviour

**Figure 5.**
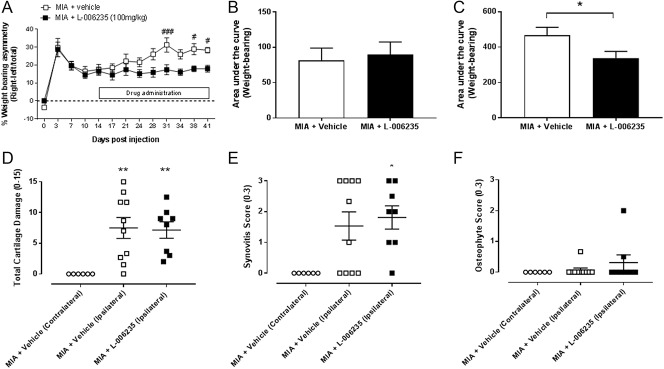
Effects of L-006235 on established MIA-induced weight-bearing asymmetry (study 3). Fourteen days after intra-articular injection of MIA rats received oral vehicle or L-006235 (100 mg/kg) twice daily until day 40 (n = 10 per group). Data are presented as a time course (A) and area under the curve analysis of the 14 days before (B) and 26 days after treatment (C). Group sizes were MIA + vehicle, n = 10 and MIA + L-006235, n = 10. Time course data were analysed with a 2-way ANOVA with a Bonferroni post hoc test: #*P* < 0.05, ###*P* < 0.001 MIA + vehicle vs MIA + L-006235. Area under the curve data were analysed with a *t* test *P < 0.05. Data are presented as mean ± SEM. (D–F) L-006235 treatment did not alter MIA-induced cartilage damage (D) or synovitis score (E) at day 41 after MIA injection. There was no change in osteophyte score (F) and effects of L-006235 could not be determined. Group sizes were MIA + vehicle (contralateral), n = 6; MIA + vehicle (ipsilateral), n = 10; and MIA + L-006235 (100 mg/kg) (ipsilateral), n = 8). Statistical comparison of groups used a Kruskal–Wallis test with post hoc Dunn's comparison: *P < 0.05, **P < 0.01 vs contralateral knee. Data are presented as mean ± SEM. ANOVA, analysis of variance.

In the final study, we investigated whether L-006235 (100 mg/kg) altered established pain behaviour in the MIA model. Time course data analysis revealed that therapeutic dosing of L-006235 (100 mg/kg) had a significant inhibitory effect on weight-bearing asymmetry, compared with MIA vehicle rats (Fig. 5A), but not on hind PWTs (supplementary Figure 4, available at http://links.lww.com/PR9/A32). Area under the curve analysis of weight-bearing asymmetry confirmed comparable weight bearing asymmetry in the 2 groups of MIA rats before L-006235 treatment (Fig. 5B) and a significant inhibitory effect of L-006235 over the period of treatment in MIA rats (Fig. 5C). At the final time point that pain behaviour was assessed (day 41), cartilage and synovitis score (Figs. 5D and E, supplementary Figure 5, available at http://links.lww.com/PR9/A32) in MIA-treated rats were consistent with levels at day 28 (Figs. 4A and B) and were significant compared with the contralateral joint. However, the osteophyte score at day 41 was lower than that evident at day 28 and not significant compared with the contralateral joint (Fig. 5F). Treatment with L-006235 from day 14 to 41 after MIA injection did not alter any of the joint features studied (Figs. 5D–F).

### 3.3. Bioanalysis of L-006235 plasma concentrations

**Figure 6.**
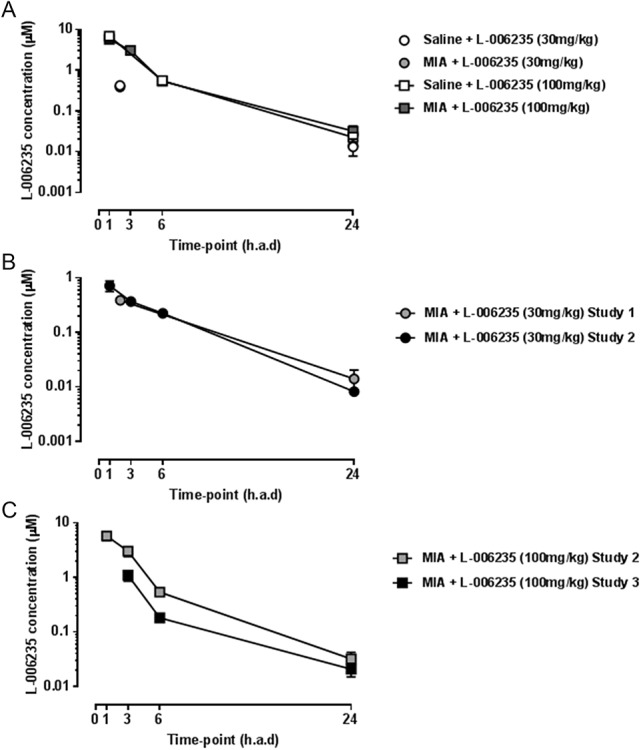
Plasma concentrations of L-006235 (µM) at 1, 3, 6, and 24 hours after dose (h.a.d). Data were collected 8 and 22 days after MIA induction in the first study, 9 and 27 days after MIA induction in the second study, and 40/41 days after MIA induction in the third study. (A) Plasma concentrations of L-006235 after dosing with 30 and 100 mg/kg in MIA- or saline-injected rats. The results for the 30- and 100-mg/kg doses are from the first and second preventative study, respectively. (B) Plasma concentrations after dosing with 30 mg/kg L-006235 in the first (gray circles) and second (black circles) preventative study. (C) Plasma concentrations after dosing with 100-mg/kg L-006235 in the second preventative study (gray squares) and in the therapeutic study (black squares). Data are presented as mean ± SEM.

Plasma concentrations of L-006235 were assessed in the 3 studies to confirm exposure to L-006235 between the studies. The exposure to L-006235 was similar in MIA- and saline-injected rats for both preventative doses studied (Fig. 6A). Similar exposures were observed between the 2 preventative studies after administration of L-006235 at 30 mg/kg twice daily (Fig. 6B). The exposure observed after administration of L-006235 at 100 mg/kg twice daily in the therapeutic study tended to be lower than the exposure observed in the preventative study (Fig. 6C). The mean PK parameters were calculated from the second preventative study and are shown in Table 2. There was a more than dose-proportional increase in exposure when increasing the dose from 30 to 100 mg/kg (Table 2).

**Table 2:**
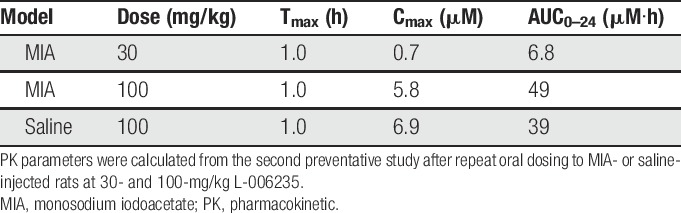
Mean PK parameters for L-006235.

### 3.4. L-006235 attenuates serum CTX-I and II levels

**Figure 7.**
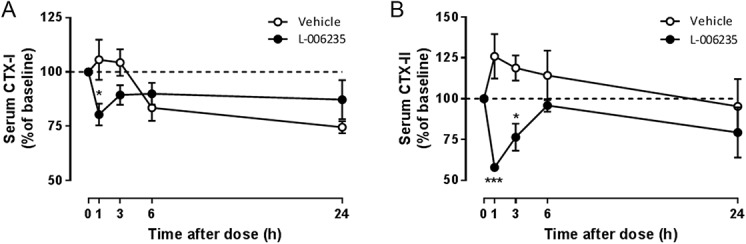
Effect of L-006235 on serum CTX-I (A) and CTX-II (B) levels after a single dose of 100 mg/kg to naive rats. Data were analysed with a 2-way ANOVA followed by Bonferroni multiple comparison test: *P < 0.05. ***P < 0.001 L-006235 vs vehicle. Data are presented as mean ± SEM (n = 3/group). ANOVA, analysis of variance.

Treatment of naïve rats with L-006235 (100 mg/kg) significantly reduced the serum CTX-I levels at 1 hour after dose by 20%, compared with baseline (Fig. 7A). In vehicle-treated naïve rats, serum CTX-I levels were instead increased by 6% at 1 hour, compared with baseline. The reduction in CTX-I levels by L-006235 was statistically significant vs baseline (*P* < 0.05) and vs vehicle (*P* < 0.05). No significant reductions in CTX-I were observed at 3 hours after L-006235 administration. Serum CTX-I levels are known to vary on food intake, and thus the results at 6 and 24 hours after dose (when the animals were allowed food again) were not conclusive. Similarly, serum CTX-II levels were reduced in L-006235–treated rats at 1 hour (by 42%) and 3 hours after dose (by 24%), compared with baseline (Fig. 7B). The reductions were statistically significant vs baseline (*P* < 0.05) at 1 hour and statistically significant vs vehicle at 1 hour (*P* < 0.001) and 3 hours (*P* < 0.05). The CTX-II levels returned to baseline at 6 hours after dose. In vehicle-treated rats, serum CTX-II levels were increased by 26% and 19%, compared with baseline, at 1 hour and 3 hours, respectively. Overall, the results indicate a significant, but transient in vivo target engagement of cathepsin K by L-006235.

## 4. Discussion

This study reports for the first time the beneficial effects of a cathepsin K inhibitor on the development of pain behaviour in a well-established rodent model of OA pain. Oral treatment with the cathepsin K inhibitor L-006235 significantly attenuated the development of pain behaviour in the MIA model of OA pain. Inhibitory effects of L-006235 were most evident on weight-bearing asymmetry, which was significantly attenuated from day 14 and then maintained for the duration of the study. Only the higher dose of preventative L-006235 significantly blocked the lowering of hind PWTs at the final 2 time points of the study. Therapeutic treatment with L-006235 from 14 days after induction of the MIA model significantly prevented further changes in weight-bearing asymmetry; however, L-006235 did not significantly reverse the already established weight-bearing asymmetry. In addition, therapeutic treatment with L-006235 did not alter lowered hind PWTs in MIA rats, compared with vehicle. Pharmacokinetic analysis confirmed that the exposure to L-006235 was higher at the 100-mg/kg dose, compared with the 30-mg/kg dose.

The finding that L-006235 attenuates weight-bearing asymmetry in the MIA model is consistent with an earlier report that 1-month administration of another cathepsin K inhibitor (AZ12606133) reduced mechanosensitivity of joint afferent fibres to non-noxious and noxious movement in the Dunkin–Hartley guinea pig model of spontaneous OA.^29^ In both our behavioural study and the earlier electophysiological study, the effects of the cathepsin K inhibitor were relatively slow in onset suggestive of mechanisms other than direct antinociceptive effects on the sensory nerve responses. In our study, we also assessed potential effects of L-006235 on spinal mechanisms of central sensitization, which are known to accompany the lowered hind PWTs in the MIA model. Previously, we have reported an increase in the expression of GFAP in the dorsal horn of the spinal cord,^35^ which is an established marker of central sensitization.^9^ In this study, the higher dose of L-006235 (100 mg/kg) did not significantly alter GFAP immunofluorescence in the dorsal horn of the spinal cord, suggestive of a lack of effect on at least some aspects of central sensitization that may point to a more peripheral site of action.

Previous studies have focused on the effects of cathepsin K inhibition on joint structure rather than functional pain responses. Although the association between joint damage and pain responses is weak, there is a significant association between synovitis and human OA pain.^38^ In this study, the doses and treatment schedule used with L-006235 did not significantly alter cartilage damage, synovitis score, or osteophyte score in the MIA model. Regression analysis did reveal an effect of L-006235 on the relationship between changes in weight-bearing and synovitis score, which may point to an action of L-006235 on inflammatory pain mechanisms at the end of the study. Treated animals displayed less pain behaviour than would be predicted by their synovitis score, suggesting that L-006235 might reduce the pain caused by inflammation. A limitation of our study is that we did not investigate the potential effects of the treatment on the immune response, which may then influence the pain behaviour. To further explore possible direct effects of L-006235 on the inflammatory pain response at the end of the study, immunohistochemical analysis of knee joint sections quantified the numbers of CD68^+^ and CD206^+^ macrophages in the synovium of MIA vehicle vs MIA L-006235–treated rats. However, these studies were inconclusive, and we found no evidence for a significant increase in either population of cells in the synovium in the MIA model and no further change by the treatment. It is feasible that this analysis was underpowered, lack of existing data on these populations of cells in this model limited our ability to perform a power calculation. Bone marrow macrophages are known to express cathepsin K and have been implicated in tumour progression in bone.^15^ The contribution of macrophages in the bone to OA pain is unknown; it is feasible that these cells, or other cell types within the synovium, are a direct or indirect target of this treatment and underpin the inhibitory effects on pain behaviour. Studies using imaging methods such as SPECT/CT to study-activated macrophage involvement may shed light on the mechanisms of action of cathepsin K inhibitors in the future.^45^

Cathepsin K is expressed by osteoclasts and has an essential role in bone resorption in human and mice,^1^ and overexpression is associated with spontaneous synovitis and cartilage degeneration.^30,33^ Our study of the effects of L-006235 was based on the mounting evidence of a contribution of osteoclasts to OA pain behaviour. In previous work, effects of cathepsin K inhibition on bone resorption were not associated with changes in osteoclastogenesis or survival of osteoclasts,^24,28^ and therefore, the effects of L-006235 on the number of osteoclasts were not quantified in this study. Comparison of the different classes of drugs, which modulate osteoclast function on pain behaviour and joint pathology in models of OA and inflammatory arthritis, highlights mechanistic differences. Unlike the bisphosphonates, cathepsin K inhibitors attenuate osteoclastic activity without altering numbers of osteoclasts and do not alter bone formation.^39^ Bisphosphonates have beneficial effects on joint pathology and pain behaviour in models of OA. In our previous study, effects of preventative treatment with zoledronate on pain behaviour in the MIA model were comparable in magnitude with the effects of L-006235; however, therapeutic treatment was not studied.^37^ Significant preventative, and reversal, effects of zoledronate on pain behaviour in MIA rats were reported in a separate study.^40^ The time course of the effects of zoledronate on pain behaviour was similar to those of L-006235 in this study. However, there were marked differences in terms of joint endpoints; at early time points zoledronate inhibited osteoclast-mediated cartilage resorption, and at later stages of the model treatment improved subchondral bone and cartilage integrity but not synovitis.^40^ The bisphosphonate alendronate inhibited bone resorption and had chondroprotective effects in a surgical model of OA, although pain was not analysed.^13^

Previously, we reported beneficial effects of a modified version of osteoprotegerin (OPG), which acts to sequester receptor activator of nuclear factor kappa-Β ligand and inhibits the number of subchondral bone osteoclasts, in the MIA model of OA pain.^37^ Osteoprotegerin-Fc prevented pain behaviour in the MIA model, effects accompanied by a robust inhibition in cartilage damage and synovial inflammation, and bone features of OA (osteoclast number and osteophyte score).^36^ The magnitude of inhibitory effects of preventative L-006235 on pain behaviour in the MIA model presented herein were comparable with the effects of OPG-Fc.^37^ In addition, the ability of L-006235 to halt further progression of pain behaviour in the MIA model once treatment commenced was also consistent with the therapeutic effects of OPG-Fc in this model. However, unlike OPG-Fc, L-006235 did not alter osteophyte score in the MIA model. The findings presented herein are consistent with the lack of effect of L-006235 on bone erosion in a model of inflammatory arthritis,^41^ which contrast the beneficial effects of OPG-Fc on this feature in the inflammatory arthritis model.^34^ Overall, the differences in the effects of OPG-Fc vs L-006235 on joint features in these models of 2 different types of arthritis suggest that mechanisms additional to cathepsin K also contribute to changes in joint structure.

To date, most previous studies in OA models have focused on the potential beneficial effects of cathepsin K inhibitors on cartilage pathology. In the anterior cruciate ligament transection (ACLT) model of OA in the rabbit L-006235 (50 mg/kg/d for 7 weeks from 1 week after surgery) significantly lowered urine CTX-II levels at 3 weeks after treatment, an effect maintained until the end of the study.^14^ After 7 weeks of L-006235 treatment, the Mankin score of joint pathology was significantly lower and bone features were improved, compared with vehicle-treated rabbits.^14^ Consistent with this finding, murine cathepsin K deletion had chondroprotective effects in the ACLT model of OA compared with wild-type controls; however, there were no beneficial effects on osteophyte formation.^14^ Positive effects of 28 day treatment with the cathepsin K inhibitor SB-553484 on cartilage damage in a canine model of OA have also been reported.^6^ Similarly, prophylactic and therapeutic effects of the potent and selective cathepsin K inhibitor MIV-711 on joint pathology in the rabbit ACLT and dog partial medial meniscectomy models were also recently reported.^26^ In the rabbit ACLT model, MIV-711 was given once daily for 7 weeks, starting 1 week after surgery (similar protocol as for in the L-006235 study above) and in the dog partial medial meniscectomy model for 28 days, starting 1 day before surgery (similar protocol as for the SB-553484 study above).^26^ The effects of L-006235 on histopathology in the collagen-induced arthritis model in mice were also evaluated in both prophylactic and therapeutic settings after dosing with L-006235 at 25 mg/kg/d. Both cartilage and bone damage, and clinical score, were significantly attenuated by preventative L-006235, whereas a therapeutic treatment once clinical signs of disease were established did not have significant effects.^41^ On the basis of these previous studies, our PK data and the engagement of L-006235 with cathepsin K reported herein, it seems that the duration of treatment, and dose used in our study (ie, up to 100 mg/kg twice daily for 28 days) was sufficient to attenuate pain behaviour but not structural changes to the joint in this model.

Our demonstration that the cathepsin K inhibitor attenuates the development, and halts established, weight-bearing asymmetry in the MIA model of OA pain supports the further investigation of the analgesic potential of this class of drugs as well as their effects on joint structure. Although our univariate analysis indicated that L-006235 altered the association between pain behaviour and synovitis, this is likely mediated by a cell type other than CD68^+^ or CD206^+^ macrophages. It is feasible that the positive effects of L-006235 on pain behaviour arise due to off-target effects. However, the inhibitory effect of L-006235 on human cathepsin S is 8 to 10 µM, and on mouse cathepsin S is 2.4 µM,^32^ making this an unlikely target for the doses and exposures achieved in this study. Nevertheless, the lack of potency data for rat cathepsin S means that we cannot rule out a species-specific effect of L-006235. From a mechanistic perspective, the more limited effects of L-006235 on lowering of hind PWTs in this study is not consistent with the robust inhibitory effects of cathepsin S inhibition on measures of allodynia in models of neuropathic pain.^5^ Overall, our novel finding that cathepsin K inhibition is analgesic in a clinically relevant model of OA pain provides evidence for a new therapeutic target for OA pain.

## Disclosures

The authors declare no conflicts of interest.

## Appendix A. Supplemental digital content

Supplemental digital content associated with this article can be found online at http://links.lww.com/PR9/A32.
